# Manipulation Planning for Object Re-Orientation Based on Semantic Segmentation Keypoint Detection

**DOI:** 10.3390/s21072280

**Published:** 2021-03-24

**Authors:** Ching-Chang Wong, Li-Yu Yeh, Chih-Cheng Liu, Chi-Yi Tsai, Hisasuki Aoyama

**Affiliations:** 1 Department of Electrical and Computer Engineering, Tamkang University, New Taipei City 25137, Taiwan; 606470093@s06.tku.edu.tw (L.-Y.Y.); 136382@mail.tku.edu.tw (C.-C.L.); chiyi_tsai@mail.tku.edu.tw (C.-Y.T.); 2 Department of Mechanical and Intelligent Systems Engineering, University of Electro-Communications, Tokyo 182-8585, Japan; aoyamaxer@gmail.com

**Keywords:** object re-orientation, pick-and-place, Mask R-CNN, semantic segmentation, 3D keypoint detection

## Abstract

In this paper, a manipulation planning method for object re-orientation based on semantic segmentation keypoint detection is proposed for robot manipulator which is able to detect and re-orientate the randomly placed objects to a specified position and pose. There are two main parts: (1) 3D keypoint detection system; and (2) manipulation planning system for object re-orientation. In the 3D keypoint detection system, an RGB-D camera is used to obtain the information of the environment and can generate 3D keypoints of the target object as inputs to represent its corresponding position and pose. This process simplifies the 3D model representation so that the manipulation planning for object re-orientation can be executed in a category-level manner by adding various training data of the object in the training phase. In addition, 3D suction points in both the object’s current and expected poses are also generated as the inputs of the next operation stage. During the next stage, Mask Region-Convolutional Neural Network (Mask R-CNN) algorithm is used for preliminary object detection and object image. The highest confidence index image is selected as the input of the semantic segmentation system in order to classify each pixel in the picture for the corresponding pack unit of the object. In addition, after using a convolutional neural network for semantic segmentation, the Conditional Random Fields (CRFs) method is used to perform several iterations to obtain a more accurate result of object recognition. When the target object is segmented into the pack units of image process, the center position of each pack unit can be obtained. Then, a normal vector of each pack unit’s center points is generated by the depth image information and pose of the object, which can be obtained by connecting the center points of each pack unit. In the manipulation planning system for object re-orientation, the pose of the object and the normal vector of each pack unit are first converted into the working coordinate system of the robot manipulator. Then, according to the current and expected pose of the object, the spherical linear interpolation (Slerp) algorithm is used to generate a series of movements in the workspace for object re-orientation on the robot manipulator. In addition, the pose of the object is adjusted on the z-axis of the object’s geodetic coordinate system based on the image features on the surface of the object, so that the pose of the placed object can approach the desired pose. Finally, a robot manipulator and a vacuum suction cup made by the laboratory are used to verify that the proposed system can indeed complete the planned task of object re-orientation.

## 1. Introduction

With the development of intelligent automation and artificial intelligence technologies such as deep learning, applications and development of intelligent robots have gradually attracted attention in the academic and industrial fields. Among the application requirements of many robots, the development of service robots has become one of the important projects. For example, for a service robot used in stores, if the robot is used to assist service personnel in placing and sorting goods on shelves, key technologies such as image recognition, object picking strategies, and collision avoidance are needed for the robot. The appearance, weight, size, and placement relationship of each product directly affect the complexity of image recognition, picking strategies, and mechanisms design. Therefore, how to determine the object’s pick-and-place method for robot manipulators is worthy of discussion.

The pick-and-place tasks in robotics have been extensively developed and researched not only in industrial manufacturing but also in academia. In recent years, according to the rise of deep neural networks, in addition to classify and logistic regression [1,2], deep learning has also been widely used in the pick-and-place tasks for robot manipulators. It can be roughly divided into four topics: (i) object detection; (ii) object pose estimation; (iii) object picking planning; and (iv) robot motion planning. Regarding object detection, there has some research on the architecture of neural network used for object detection in recent years, such as Region-based Convolutional Neural Networks (R-CNN) related series [3,4,5,6], You Only Look Once (YOLO) series [7,8,9], and Single Shot MultiBox Detector (SSD) [10]. The R-CNN series use a two-stage method to first extract candidate Region Of Interest (ROI) and then classify each ROI so that they can achieve very high accuracy. YOLO series and SSD are one-stage methods, using deep neural network to simultaneously classify objects and detect object positions. The one-stage methods are usually faster and suitable for real-time applications, but the overall recognition accuracy may be lower than the two-stage methods. Object pose estimation is commonly based on RGB-D image [11] or 3D model [12,13] to calculate the 3D pose of object. Manuelli et al. [14] proposed the keypoint [15] method, which is used to detect human skeleton and find the relative keypoints, and combined it with local dense geometric information from a point cloud. In this way, a simpler pose vector can be used to represent the object pose in the environment and facilitate subsequent object picking operations. Semochkin et al. [16] proposed a keypoint detection method for different camera perspectives based on a trained CNN and used keypoints as the representation of object in the task of grasping task. Vecerik et al. [17] proposed a self-supervised training system to detect 2D keypoints for all individual scene views and used CNN to estimate the possible depth of objects, so as to estimate the 3D location of each keypoint.

Research in robotic manipulation over the past decades has mostly focused on how to pick up objects [18], but it has seldom discussed how to manipulate objects or the process of placing objects after being grasped. In fact, in the research about robot pick-and-place planning, most of the scenarios are drop the object down from a height to the box or table without considering the consequences [19,20,21]. There has not yet been a state-of-mind research for the object placement task, so it has been deriving the issue of multiple regrasp [22,23,24] to achieve the issue of specific placement or the issue of re-orientation [25] of the object. In recent years, there have been related studies on generating grasping strategies based on how to use the grasped objects [18]. For example, Do et al. [26] used the affordance of object for task-oriented grasping, while Lai et al. [27] and Qin et at. [28] used the grasped objects as the robot manipulator’s tool to achieve operation control according to the specific objectives.

The purpose of this paper is to combine object detection, object pose estimation, and motion planning for the robot manipulator to achieve the function of object re-orientation. However, since the method of directly segmenting the target object in the 3D point cloud needs to build a 3D model for each target object independently, it also cannot achieve the characteristics that are widely used in the same type of object. As a result, this paper starts from 2D image recognition which is based on the deep learning method, combined with the depth image, and converts the pixel coordinates of the segmented area into 3D coordinates to complete the task of object pose estimation. It also integrates functions such as coordinate conversion, grasp planning, and trajectory planning, so that the robot manipulator can complete the object re-orientation.

The rest of this paper is organized as follows. In Section 2, the system structure of the proposed object re-orientation manipulation planning method is described. In Section 3, five main parts of the proposed 3D keypoint detection system are described. In Section 4, the object pose comparison and robot operation of the proposed manipulation planning system are described. In Section 5, some experimental results are presented to illustrate the proposed method and a laboratory-made robot manipulator is used to verify that the proposed method can indeed run on the actual robot. Finally, the conclusions are summarized in Section 6.

## 2. System Structure

**Figure 1:**
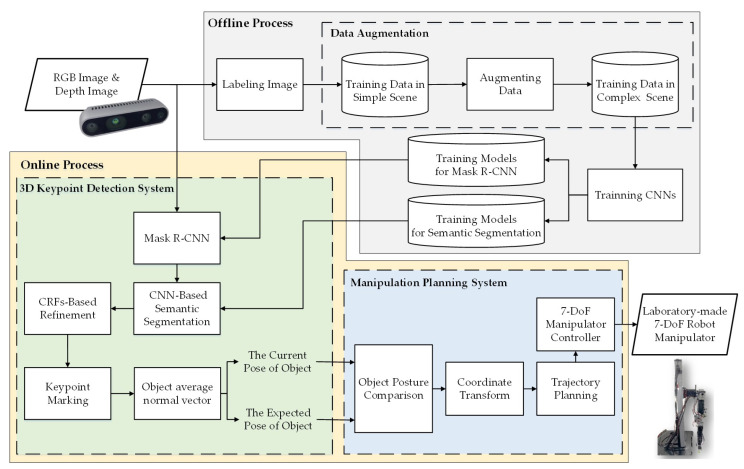
System architecture of the proposed manipulation planning method for object re-orientation.

The system architecture of the proposed manipulation planning method for object re-orientation in a category-level manner is shown in Figure 1. There are two main modules: 3D keypoint detection and manipulation planning. In the 3D keypoint detection module, since this paper uses Deep Convolutional Neural Network (DCNN) to detect the target object and each pack unit of the object, it usually requires plenty of time to build such a large training dataset to optimize the DCNN model. To solve this problem, a data augmentation module is added in the system and is used off-line to construct training dataset, which creates ample training data by superimposing a small number of manually marked datasets on different backgrounds. As a result, the database can be efficiently used to optimize the DCNN model and fine-tune a pre-train model. The data augmentation system process is introduced in detail in Section 3.5.

In the on-line process, this paper uses a trained Mask R-CNN and a CNN-based semantic segmentation for object detection. First, Mask R-CNN is used to segment the pixel level of a target object. After gaining the segmented object picture, it is input into the semantic segmentation network to segment each pack unit of the object. After obtaining the position of each pack unit of object, the centroid position of each pack unit is used as the keypoint of the object, and the 3D pose of the object is estimated through the normal vector of the keypoint. The detailed operation process is presented in Section 3 and the operating time of the real robot is described in Section 5.3.

In the manipulation planning module, the pose of the object and the normal vector of each pack unit are first converted into the working coordinate system of the robot manipulator. Then, according to the current pose and the expected pose of the object, the Slerp [29] algorithm is used to enable the robot manipulator to perform a series of movements in the workspace to re-orientate the object. Finally, the object is repositioned through the laboratory-made 7-DoF robot manipulator and vacuum suction cup. The detailed operation process is presented in Section 4.

## 3. 3D Keypoint Detection System

The main purpose of this section is to introduce how to establish the 3D keypoint detection system and explain how to integrate multiple object detection algorithms to achieve the purpose of estimating pose of the object. There are five main parts: (a) Mask R-CNN; (b) CNN-based semantic segmentation; (c) CRFs-based refinement; (d) keypoint annotation and object average normal vector; and (e) data augmentation for training dataset generation. They are described as follows.

### 3.1. Mask R-CNN

**Figure 2:**
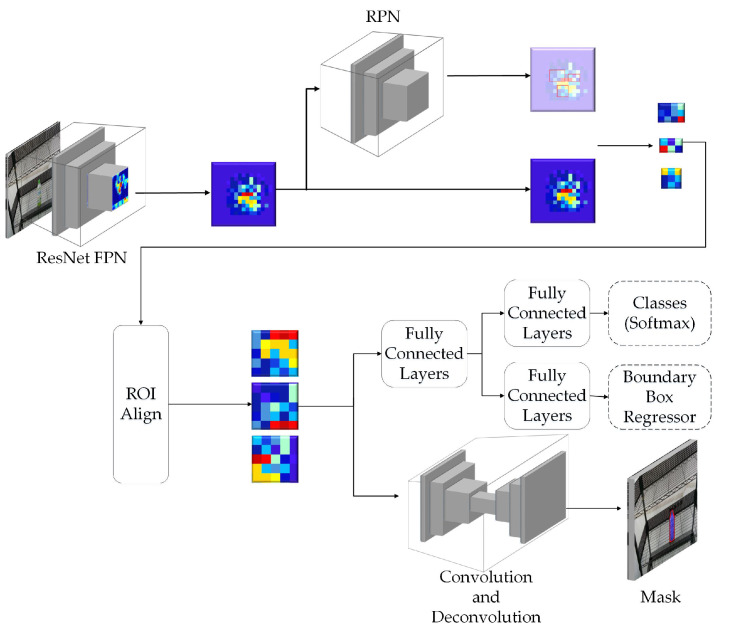
Detailed two-stage structure of Mask R-CNN architecture [6].

Mask R-CNN, a two-stage object detection algorithm which can achieve category-level detection and a mask that delimits the pixels constituting each object, enables the complete contour pixel position of the object to be accurately segmented while maintaining the operating speed. This algorithm is a deep learning algorithm based on Faster R-CNN [5] proposed by He et al. [6] for object detection and instance segmentation. The two-stage structure of Faster R-CNN model is used for object detection. In the first stage, Feature Pyramid Network (FPN) is used to generate a set of regions of interest as potential bounding box candidates to improve the robustness of detecting objects of different sizes. In the second stage, the candidate regions of interest are classified, and then the pixel position of the object is predicted by deconvolution, which provides three outputs for each object: a class label, a bounding box that delimits the object, and a mask that delimits the pixels that constitute. The mask image with the highest confidence index is selected as the input of the semantic segmentation system. Figure 2 shows the detailed two-stage architecture of Mask R-CNN.

### 3.2. CNN-Based Semantic Segmentation

**Figure 3:**
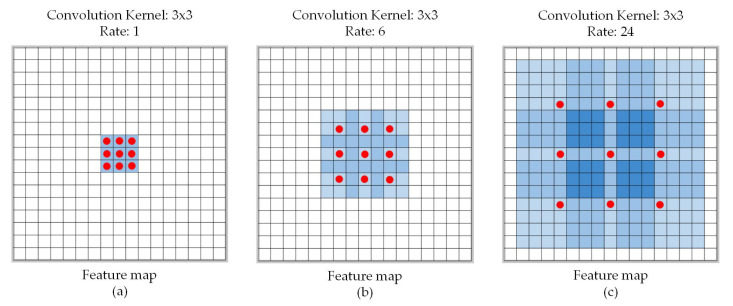
Examples of atrous convolution [30].

This paper uses the semantic segmentation method based on convolutional neural network (CNN-based semantic segmentation) to perform secondary segmentation on the complete object mask image output in the previous section. The purpose is to segment the precise position of each pack unit of the object. For example, the PET bottle can split into two pack units: the body and the cap. In this paper, the existing DeepLab-ResNet [30] is employed as the network architecture at this stage, which is characterized by the use of atrous convolution shown in Figure 3 for convolution processing. The benefit is that it can maintain a larger Field-of-View (FOV) after processing with the convolution layer while maintaining the resolution of the feature map and the size of the convolution kernel, to ensure that more features are stored when the image size is equal during the convolution process.

### 3.3. CRFs-Based Refinement

After the CNN-based semantic segmentation process described in Section 3.2, an approximate object result can be found, which may affect the subsequent centroid calculation. To overcome this problem, a fully connected CRFs layer [31] is used to refine the CNN output, produce accurate semantic segmentation results, and recover object boundaries at a level of detail. The CRFs are a type of discriminative undirected probabilistic graphical model, which represents the Markov random field that outputs another set of random variables under the condition of a given set of random input variables *x*. The energy function of the model is described by

(1) $$E\left(x\right)=\sum_{i}\theta_{i}\left(x_{i}\right)+\sum_{ij}\theta_{ij}\left(x_{i},x_{j}\right)$$

where *x* is the label assignment for pixels, $\theta_{i}\left(x_{i}\right)=-\log P\left(x_{i}\right)$ is the unary potential, and $P(x_{i})$ is the corresponding label assignment probability at pixel *i* obtained from the CNN output. The following expression can be used to connect all pairs of image pixels, where *i*, *j* is the pairwise potential for each pair

(2) $$\theta_{ij}=\mu \left(x_{i},x_{j}\right)\left[\omega_{1}exp\left(-\frac{{∥p_{i}-p_{j}∥}^{2}}{2\sigma_{\alpha }^{2}}-\frac{{∥I_{i}-I_{j}∥}^{2}}{2\sigma_{\beta }^{2}}\right)+\omega_{2}exp\left(-\frac{{∥p_{i}-p_{j}∥}^{2}}{2\sigma_{\gamma }^{2}}\right)\right]$$

In Equation (2), $\mu (x_{i},x_{j})=1$ if $x_{i}\neq x_{j}$, while the rest are all 0, which means that only nodes with distinct labels are penalized. The two Gaussian kernels are used to express in different feature spaces. The first Gaussian kernel depends on the penalty between two pixel positions (denoted as $p_{i}$ and $p_{j}$) and between their RGB values (denoted as $I_{i}$ and $I_{j}$). The second kernel considers spatial proximity when enforcing smoothness and only depends on the pixel position.

### 3.4. Keypoint Annotation and Object Average Normal Vector

After the output of the above process, the complete contour of pack unit is marked with different colors, so that the centroid position of each part can be calculated separately. Take the bottle cap as an example. The centroid calculation method is described as follows.

**Step 1:** Convert the pack unit mask into grayscale, and define the points on the mask image by

(3) cap=[point1,point2,⋯,pointk]

where *k* is the length of pixel of the bottle cap mask.

**Step 2:** Define the maximum and minimum coordinate values in x-axis and y-axis by

(4) $$\left\{\begin{array}{c} i_{min}=Min\left(point1.x,point2.x,\ldots pointk.x\right)\\ i_{max}=Max\left(point1.x,point2.x,\ldots pointk.x\right)\\ j_{min}=Min\left(point1.y,point2.y,\ldots pointk.y\right)\\ j_{max}=Max\left(point1.y,point2.y,\ldots pointk.y\right) \end{array}\right.$$

**Step 3:** Calculate the moment of order (p+q) of an image in the interval range *x*, *y* for a 2D continuous function f(x,y) by

(5) $$m_{pq}=\sum_{x}\sum_{y}x^{p}y^{q}f\left(x,y\right)$$

where p,q=0,1,2,….

**Step 4:** Calculate the zero-order moment $m_{00}$ of the function f(x,y), which represents the total of mass of the given image and is defined as

(6) $$m_{00}=\sum_{i=i_{min}}^{i_{max}}\sum_{j=j_{min}}^{j_{max}}f\left(i,j\right)$$

**Step 5:** Calculate the two first-order moments $m_{10}$ and $m_{01}$, which represent the centre of mass of the given image and are defined as

(7) $$m_{10}=\sum_{i=i_{min}}^{i_{max}}\sum_{j=j_{min}}^{j_{max}}i\times f\left(i,j\right)$$

(8) $$m_{01}=\sum_{i=i_{min}}^{i_{max}}\sum_{j=j_{min}}^{j_{max}}j\times f\left(i,j\right)$$

**Step 6:** Calculate the centroid $\bar{x}$ and $\bar{y}$ by dividing $m_{10}$ and $m_{01}$ by $m_{00}$, which can be expressed as

(9) $$\bar{x}=\frac{m_{10}}{m_{00}},\;\bar{y}=\frac{m_{01}}{m_{00}}$$

Set the calculated centroid position to a depth of range $d_{pcl}$, calculate the vector of each pixel in the range through the point cloud image, and calculate the average normal vector of the object surface. The average normal vector calculation process is shown in Algorithm 1.

### 3.5. Data Augmentation for Training Dataset Generation

**Figure 4:**
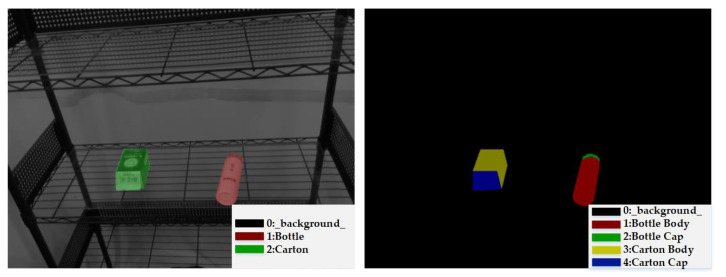
An example of training sample.

This subsection introduces the data augmentation module for deep learning proposed in this paper. In the preparation of training data, since this paper uses the Mask R-CNN to perform the first stage of object detection, its main purpose is to segment the complete object contour. As a result, the ground truth data are marked with the type of individual objects, as shown in the left picture of Figure 4. In the second stage of semantic segmentation, the main purpose is to use the complete object contour image in the output of previous stage to detect each pack unit of the object, so as to annotate the keypoints of the object in Section 3.4. The corresponding ground truth is shown in the right picture of Figure 4, each color pixel representing each pack unit of objects classified from the input image.
**Algorithm** **1:** Get average normal vector from point clouds.
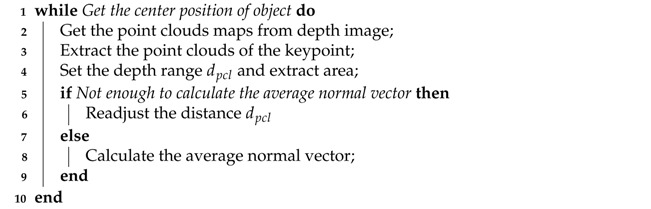



**Figure 5:**
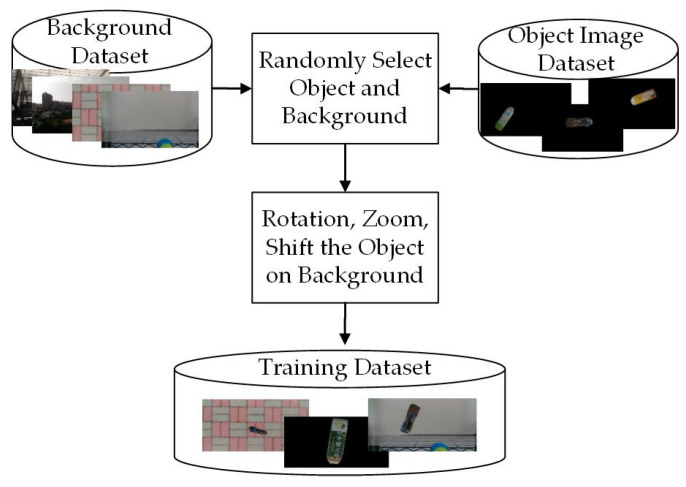
Procedure of the proposed data augmentation method.

The procedure of proposed data augmentation system is shown in Figure 5. This paper uses a variety of background pictures and manually marked images to randomly superimpose and related image processes to geometrically transform the ground truth, which includes rotation, zoom, shift, random stack, etc., so that the object can be combined with the background to synthesize a random training dataset while retaining the original features for training in DCNNs.

In the training process, this paper uses the data augmentation system to build individual datasets to train Mask R-CNN and CNN-based semantic segmentation network. Table 1 shows the information of data augmentation and network training, which includes the backbone network, the number of objects, the number of random scenes, the manually label photos, the number of individually generated training photos, and the resolution of the photos.

## 4. Manipulation Planning System

The main purpose of this section is to introduce how to use the keypoints of the object combined with the robot manipulator to plan the operation of sucking the object and explain how to compare the expected pose and current pose of object to achieve the purpose of re-orientating the object. There are two main parts: (a) object pose comparison; and (b) robot operation. They are described as follows.

### 4.1. Object Pose Comparison

After obtaining the 3D keypoints of the object in Section 3, this section mainly discusses how to use the keypoint information of each pack unit to compare the pose difference of the object. The task of “How to re-orientate a PET bottle on the shelf” is presented as an example; the keypoints $p_{target\_cap}$ and $p_{target\_bottle}$ of the pack unit are used to represent the positions of the PET bottle cap and PET bottle body, respectively. The points $p_{target\_bottle}$ and $p_{target\_cap}$ are used to obtain a unit vector $v_{target\_axis}$ to represent the pose of the PET bottle, and the point $p_{target\_cap}$ is projected on the camera coordinate of the *y*-axis to get a project point $p_{target\_cal}$. Then, the rotation angle $\theta_{obj\_rotation}$ of the PET bottle in space can be calculated by

(10) $$\theta_{obj\_rotation}=\pi -\left(a\cos\frac{\bar{p_{target\_cap}p_{target\_cal}}}{\bar{p_{target\_cap}p_{target\_bottle}}}\right)$$

After calculating the rotation angle $\theta_{obj\_rotation}$ of the object, the position of the object and the pose unit vector in the workspace can be obtained. Next, invert this pose unit vector as the orientation of end-effector to suck the object by converting the coordinate system from camera to robot so that the robot manipulator can be used to suck the target object.

**Figure 6:**
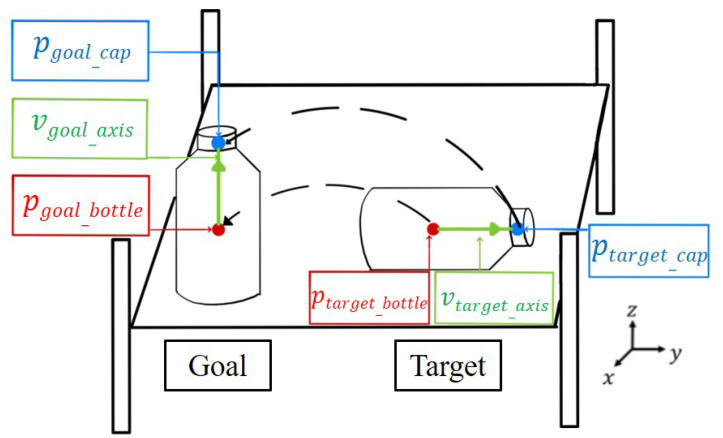
The schematic diagram of object reorientation.

Finally, by comparing the expected pose $v_{goal\_axis}$ that is initially given by the user with the current pose $v_{target\_axis}$, the initial and target suck position and orientation of the end-effector of the robot manipulator can be obtained through coordinate conversion, and the object can be sucked from the target to goal to achieve the purpose of “re-orientate a PET bottle on the shelf”. The schematic diagram is shown in Figure 6, where the black dashed line represents the movement trajectory of the pick-and-place action in workspace of two keypoints.

### 4.2. Robot Operation

**Figure 7:**
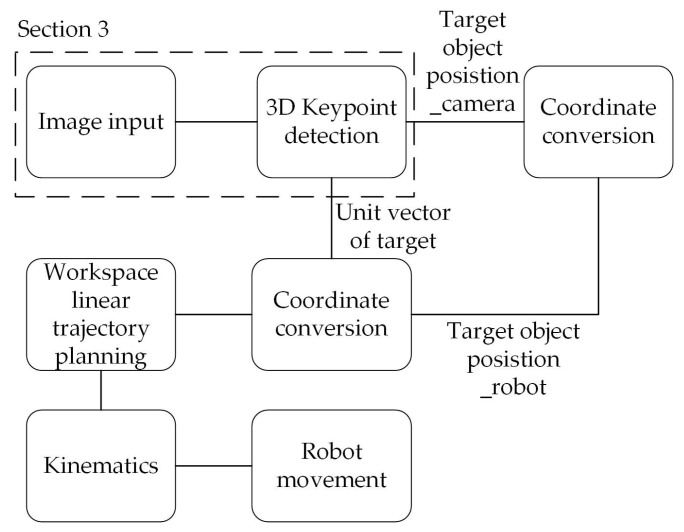
Flowchart of robot manipulator operation planning.

This section mainly discusses the coordinate conversion and the linear trajectory planning in workspace, the purpose of which is to generate the trajectory of endpoint of robot manipulator. The flowchart of robot manipulator operation planning is shown in Figure 7. The unit vector of an object can be obtained through the 3D keypoint detection in Section 3; through the coordinate conversion, an orientation of endpoint of robot to suck the object can be obtained. Finally, the operation of the robot manipulator is planned through the kinematics [32].

After obtaining the position and orientation that are represented by quaternion of the end-effector of the robot manipulator, the current pose and the target pose of the object can be calculated by interpolating the two quaternions at a ratio to plan the movement path. The purpose of this section is to use the Slerp algorithm to generate the trajectory points that the robot manipulator passes through and arrange the rotation of each point, which is expected that the trajectory of orientation to also be smooth.

To perform general linear interpolation between point $p_{1}$ and $p_{2}$, if *T* interpolation points are to be generated, the interpolation points at time *t* are calculated as

(11) $$p_{t}=p_{1}+\frac{t}{T}\left(p_{2}-p_{1}\right)$$

**Figure 8:**
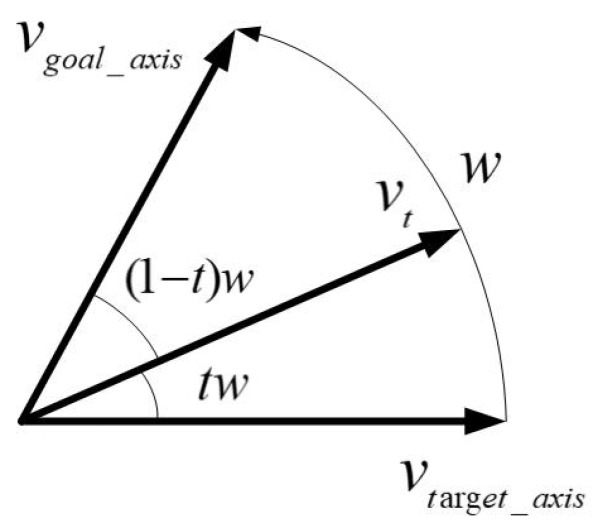
Schematic diagram of two-vector linear interpolation in a two-dimensional plane [32].

In the orientation planning part, the spherical linear interpolation of the quaternion in the four-dimensional space can be simplified to two unit vectors $v_{target\_axis}$ and $v_{goal\_axis}$ on the two-dimensional plane to interpolate on the unit circle. The purpose is that, when two endpoints of the robot manipulator are fixed, the orientation can be formed as a spherical surface in the three-dimensional space, and the trajectory between the two orientation vectors is the sphere by the approach vector of the robot manipulator. At time *t*, the angle between the two vectors is *w*, the interpolation vector $v_{t}$ is calculated by Equation (12), and the schematic diagram is shown in Figure 8.

(12) $$Slerp\left(v_{t}\right)=\frac{\sin(1-t)w}{\sin w}v_{target\_axis}+\frac{\sin tw}{\sin w}v_{goal\_axis}$$

## 5. Experimental Results

**Figure 9:**
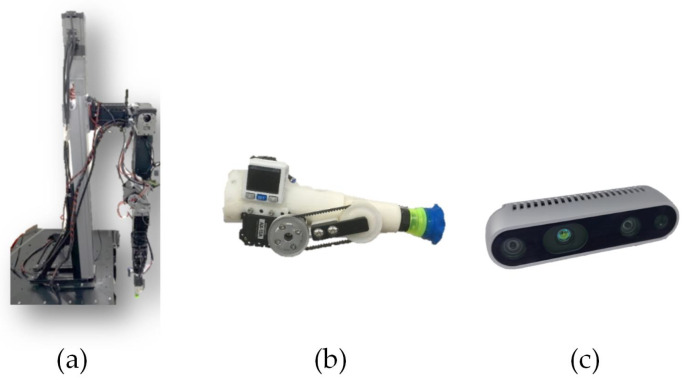
Three instruments used in the experiment: (**a**) laboratory-made 7-DoF manipulator; (**b**) laboratory-made vacuum suction cup; and (**c**) Intel® RealSense™ D435 RGB-D camera.

The purpose of this section is to verify the validity of the methods proposed in this paper. The three main hardware instruments used in the experiment are shown in Figure 9. The overall software system was implemented with Robot Operating System (ROS), which provides a standard communication tunnel to help collaborative development. The DCNN and normal vector were implemented with Tensorflow and Point Cloud Library (PCL), respectively. In this section, the experimental results of the object re-orientation are divided into three main parts: (a) image recognition results; (b) real-world demonstration; and (c) computational eficiency. They are described as follows:

### 5.1. Image Recognition Result

#### 5.1.1. CNN-Based Object Detection

Since DCNN is used to detect the target object and each pack unit of object, it usually requires a large training dataset to improve the accuracy of the method. To solve this problem, the data augmentation module described in Section 3.5 was added to the system. The information of data augmentation and network training of Mask R-CNN and CNN-based semantic segmentation is shown in Table 1. The Intersection-over-Union (IoU) metric was added to quantitatively evaluate the accuracy of the proposed visual perception algorithm. It is defined by

(13) $$IoU\left(A,B\right)=\frac{AreaofOverlap}{AreaofUnion}=\frac{\left|A\cap B\right|}{\left|A|+|B|-|A\cap B\right|}\times 100\%$$

Regarding to image recognition result, the original camera image was the input of the Mask R-CNN, and a single object was extracted to be the input of the CNN-based semantic segmentation for the pack unit detection. After that, the semantic segmentation result was optimized by the CRFs-based refinement method. The image processing results of three types of objects by the proposed method are described in Table 2 and Table 3. From the output results of the processes by the Mask R-CNN, object extraction, CNN-based semantic segmentation, and CRFs-based refinement, the mean IoU of Mask R-CNN and pack-unit segment through the comparison with ground truth data are 0.942 and 0.913, respectively. Through the experimental results, it can be verified that the proposed method can completely segment each position of pack-unit.

#### 5.1.2. Object Pose Estimation

**Figure 10:**
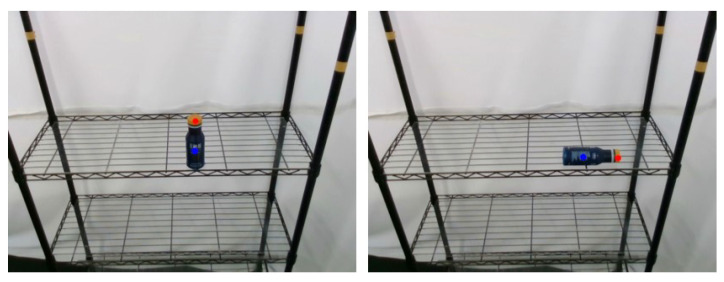
Keypoint detection results of PET bottle obtained by the proposed object pose estimation method.

Through the pack unit image output in Section 3.3, the keypoint annotation method described in Section 3.4 could be used to separately calculate the keypoints representing pack unit. The experimental results to mark the keypoints in the original image are shown in Figure 10, where the left picture is the PET bottle stand upright on the shelf and the right picture is placed flat on the shelf. The keypoints of the cap and body of the PET bottle are marked with red and blue, respectively.

**Figure 11:**
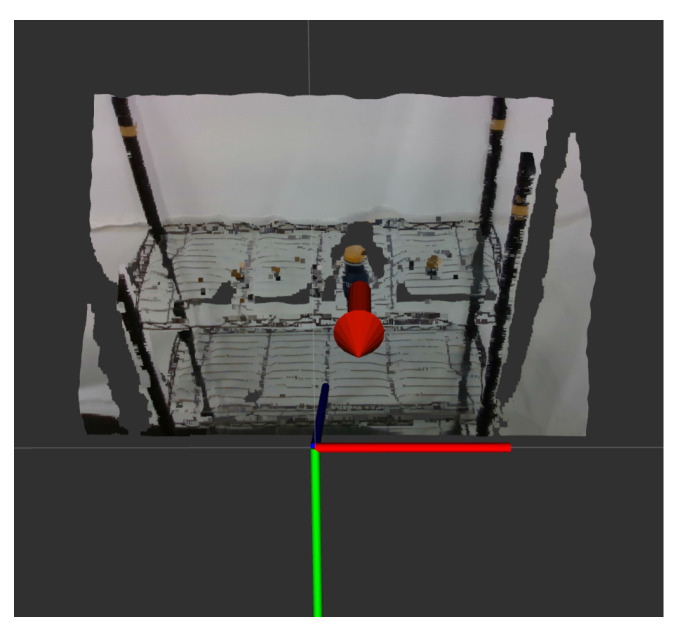
Schematic diagram of unit vector calculation results for PET bottle.

After obtaining the keypoint information of the object, through the point cloud-based unit vector calculation process described in Section 3.4, a unit vector could be obtained to represent the pose information of the object in the environment. In Figure 11, the unit vector in the workspace is described by the red arrow and the coordinate system is the coordinate system of the camera, which is installed on top of the slide rail of robot. After obtaining this unit vector, the inverse unit vector of this vector could be used as the orientation of the end-effector of the robot manipulator to suck the object through coordinate conversion, so that the robot manipulator can suck the target object.

**Figure 12:**
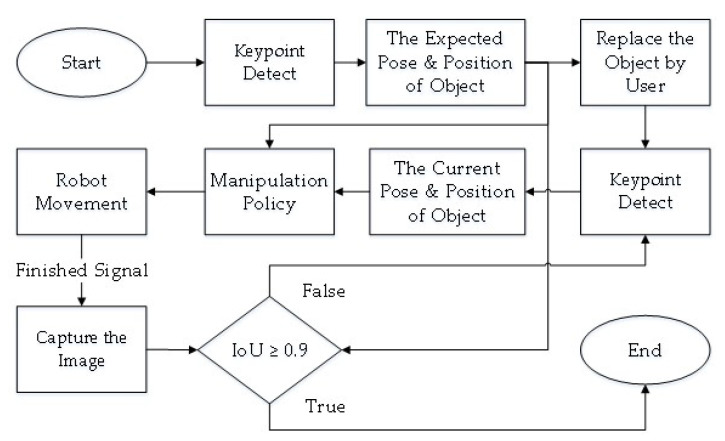
Flow chart of the experiment of the proposed manipulation planning for the object re-orientation.

To analyze the accuracy of the proposed 3D keypoint detection system, this study used the Mean Absolute Error (MAE) metric to measure the pose estimation results. Using manually marked key-points as ground truth data, the two poses of the PET bottle in Figure 12 were detected multiple times and the position and pose were transformed into the robot base frame. To measure pose estimation errors of the proposed method, the position and rotation estimation errors are, respectively, defined as

(14) $$\delta T_{\Omega }=T_{\Omega }-{\hat{T}}_{\Omega }\;and\;\delta R_{\Omega }=R_{\Omega }-{\hat{R}}_{\Omega }$$

where $\Omega =\left\{x,y,z\right\}$ represents one of the three axes of the Cartesian coordinate system. $T_{\Omega }$ and $R_{\Omega }$ represent the position and rotation of the ground truth, respectively. ${\hat{T}}_{\Omega }$ and ${\hat{R}}_{\Omega }$ represent the position and rotation of the estimate data, respectively. Based on the above definition, the mean absolute error metric is defined as

(15) $$MAE\left(\delta X_{\Omega }\right)=\frac{1}{N}\sum \left|\delta X_{\Omega }\right|$$

where *N* is the total test number and *X* represents one of the estimate variables *T* or *R*, and *N* is the total test number. The MAE measures of the pose estimation results are described in Table 4.

In Table 4, the average errors of the position estimation results obtained from the proposed method are, respectively, 0.13, 0.58, and 0.73 cm along the x-, y-, and z-axis, and the average rotation errors are, respectively, 3.27, 5.68, and 2.36 degrees across the x-, y-, and z-axis. This accuracy level is suitable for the robot manipulator to perform the object picking and re-orientation task. Therefore, the experimental results validate the pose estimation accuracy of the proposed algorithm.

### 5.2. Real-World Demonstration

**Figure 13:**
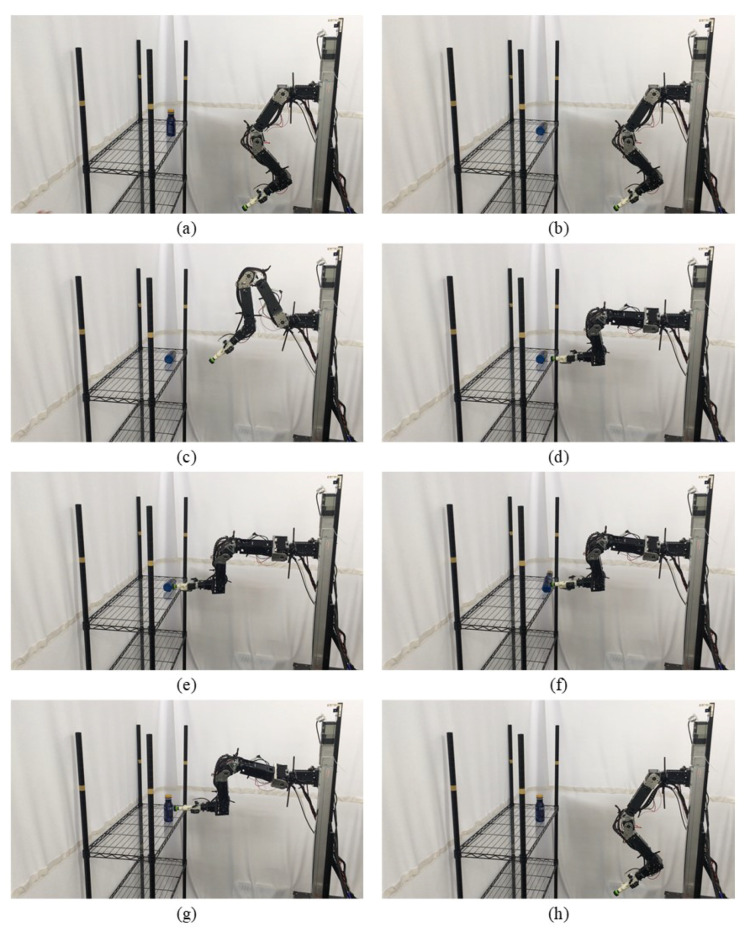
Experimental snapshots of the experiment of the proposed manipulation planning for the object re-orientation.

An experiment of the proposed manipulation planning for the object re-orientation is presented and its flow chart of the whole experiment process is described in Figure 12. First, an RGB-D camera was used to capture environmental information, and the expected position and pose of the object was estimated through the keypoint detection system described in Section 3. After obtaining the expected position and pose, the object was replaced by the user, and the keypoint detection system was used to estimate current position and pose of the object. The keypoint detection system suspended detection during the operation of the robot manipulator. By comparing the difference of the expected and current position and pose, a series of movements of the robot manipulator was generated to pick the object and re-orientate it to the expected position and pose. After completing the above process, the camera was used to capture the current pose of the object, and the picture of the expected object’s pose was compared with the Intersection over Union described in Equation (12). If the similarity was greater than 0.9, the process was determined to be successful. If the similarity was less than 0.9, the object re-orientation was performed again to achieve a closer result. To ensure that the detection system has good accuracy in the operation of the real robot, the checkerboard calibration method provided by realsense™ [33] was used to calibrate the camera before the experimental process. The movement states of the robot manipulator shown in each picture of Figure 13 are described as follows:

(a) Capture the expected position and pose of the object.
(b) Capture the current position and pose of the object.
(c) Robot initial pose.
(d) Move to the front of the object.
(e) Suck the object.
(f) Re-orientate the object.
(g) Place the object to the expected pose.
(h) Return to the initial state.

### 5.3. Computational Efficiency

The proposed system was implemented in C++ and Python running on an Ubuntu 16.04 platform personal computer that is equipped with an Intel® Core™ i7-7700 CPU, 32 GB DDR4 system memory, and a NVIDIA GeForce GTX 1080 GPU with 8 GB frame buffer memory. The average processing time in each phase of the proposed 3D keypoint detection system is described in Table 5. We can see that the average total processing time took about 1.742 s in the real operation, and the CNN-based semantic segmentation and CRFs-based refinement spent more time in the whole process.

## 6. Conclusions

An object re-orientation planning method based on 3D keypoint detection is proposed for the robot manipulator so that it can re-orientate an object from an arbitrary pose to a specified position and pose. There are three main contributions of this research: (i) In the object pose estimation system, the CNN-based object detection algorithm is used to recognize the position of each object by separating them into some pack units, and the depth image is added to estimate the object’s pose in the environment. Due to the characteristics of the CNN-based object detection algorithm, it can be executed in a category-level manner by adding several relative categories of the object, without the need to relatively build a 3D model of the object in the training phase. Compared with other 3D model-based object detection systems, this method can simplify the procedure more effectively. (ii) In the second stage of CNN-based pack unit detection, the mask image obtained by instance segmentation in the first stage is used as input so that the environmental effect is reduced and the contour of each pack unit can be accurately and completely segmented. In addition, it can simultaneously generate fewer label categories while using CRF for individual contour optimization. (iii) In the manipulation planning for object re-orientation, the spherical linear interpolation method is used to effectively generate a series of movements in the workspace for the robot manipulator by comparing the difference of the current pose and the expected pose of the object. It can be concluded from the experimental results that the proposed method can indeed complete the object re-orientation.

## Figures and Tables

**Table 1:** Information of data augmentation and network training of Mask R-CNN and CNN-based semantic segmentation.

Item	Mask R-CNN	CNN-Based Semantic Segmentation
Backbone network	ResNet-101	ResNet-101
Number of objects	3	6
Number of random scenes	10	10
Manually label photos	45	50
Automatically generate photos	4000	6000
Resolution	640 * 480	640 * 480

**Table 2:** Image processing results of three types of obtained by the proposed CNN-based object detection method.

	PET Bottle	Carton Drink	Tomato Can
Origin picture	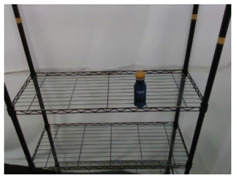	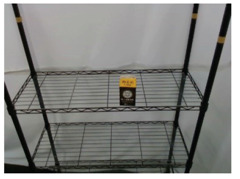	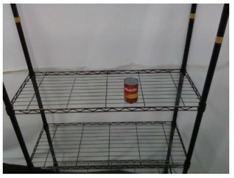
Output of Mask R-CNN	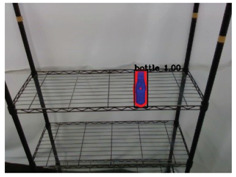	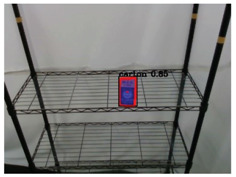	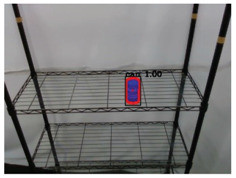
Object extraction	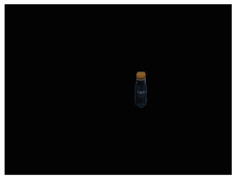	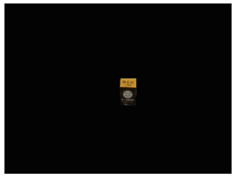	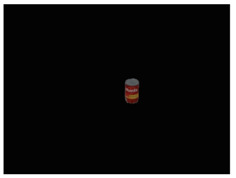
Output of CNN-based Semantic Segmentation	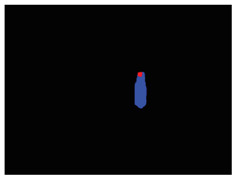	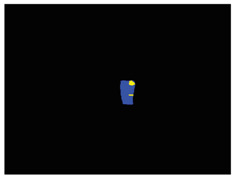	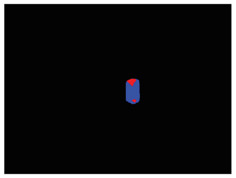
CRFs-Based refinement	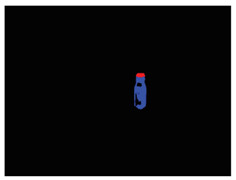	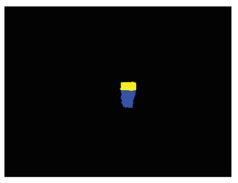	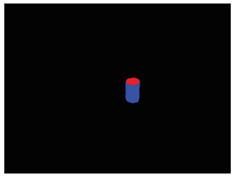

**Table 3:** Image processing results of three different pose of the PET bottle obtained by the proposed CNN-based object detection method.

	PET Bottle	PET Bottle	PET Bottle
Origin picture	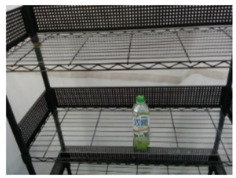	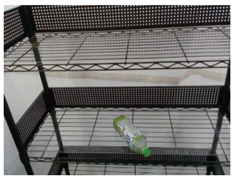	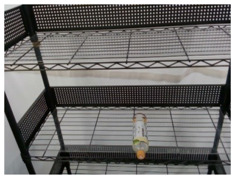
Output of Mask R-CNN	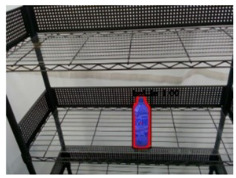	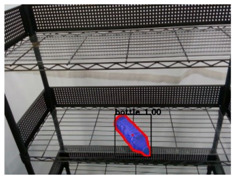	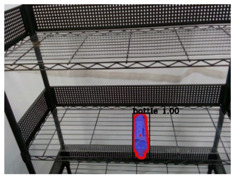
Object extraction	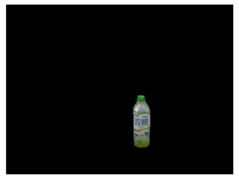	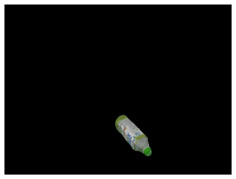	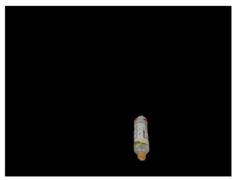
Output of CNN-based Semantic Segmentation	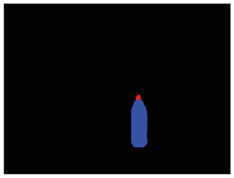	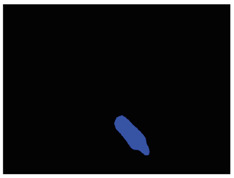	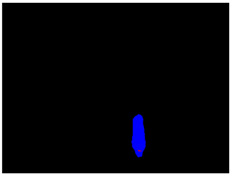
CRFs-Based refinement	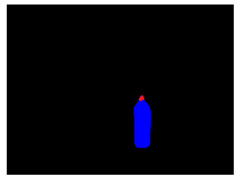	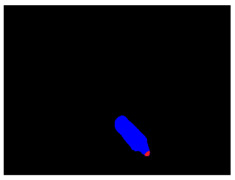	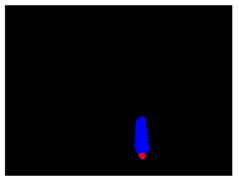

**Table 4:** MAE measures of the 3D keypoint detection system results shown in Figure 10.

MAE Value	Position Error (cm)			Rotation Error (Degree)			Total Test Number
	$\delta T_{x}$	$\delta T_{y}$	$\delta T_{z}$	$\delta R_{x}$	$\delta R_{y}$	$\delta R_{z}$	N
Figure 10 (left)	0.1426	0.6438	0.7446	3.6722	5.2038	2.7232	15
Figure 10 (right)	0.1227	0.5210	0.6892	2.8746	6.1624	1.9902	15
Total average	0.1327	0.5824	0.7319	3.2734	5.6831	2.3567	30

**Table 5:** Average processing time in each step of the proposed 3D keypoint detection system.

Function	Method	Processing Time	Proportion of Time
Visual Perception	Mask R-CNN	0.258	14.8%
	CNN-Based Semantic Segmentation	0.429	24.6%
	CRFs-Based Refinement (5 iterations)	0.996	57.1%
Object Pose Estimation	Keypoint Annotation	0.016	0.9%
	Normal Vector from Point Cloud	0.043	2.4%
Total Processing Time (in seconds)		1.742	

## Abbreviations

The following abbreviations are used in this paper:

CRFs	Conditional Random Fields
DCNN	Deep Convolutional Neural Network
FOV	Field-of-View
FPN	Feature Pyramid Network
IoU	Intersection over Union
MAE	Mean Absolute Error
PCL	Point Cloud Library
R-CNN	Region-based Convolutional Neural Network
RoI	Regions of Interest
Slerp	Spherical Linear Interpolation
SSD	Single Shot MultiBox Detector
YOLO	You Only Look Once
